# Building and Repairing the Heart: What Can We Learn from Embryonic Development?

**DOI:** 10.1155/2014/679168

**Published:** 2014-04-17

**Authors:** Ana G. Freire, Tatiana P. Resende, Perpétua Pinto-do-Ó

**Affiliations:** ^1^Instituto de Engenharia Biomédica (INEB), Universidade do Porto, Rua do Campo Alegre 823, 4150-180 Porto, Portugal; ^2^Department of Developmental and Regenerative Biology and The Black Family Stem Cell Institute, Mount Sinai School of Medicine, New York, NY 10029, USA; ^3^Faculdade de Engenharia da Universidade do Porto (FEUP), Rua Dr. Roberto Frias, s/n, 4200-465 Porto, Portugal; ^4^Instituto de Ciências Biomédicas Abel Salazar (ICBAS), Universidade do Porto, Rua de Jorge Viterbo Ferreira 228, 4050-313 Porto, Portugal

## Abstract

Mammalian heart formation is a complex morphogenetic event that depends on the correct temporal and spatial contribution of distinct cell sources. During cardiac formation, cellular specification, differentiation, and rearrangement are tightly regulated by an intricate signaling network. Over the last years, many aspects of this network have been uncovered not only due to advances in cardiac development comprehension but also due to the use of embryonic stem cells (ESCs) *in vitro* model system. Additionally, several of these pathways have been shown to be functional or reactivated in the setting of cardiac disease. Knowledge withdrawn from studying heart development, ESCs differentiation, and cardiac pathophysiology may be helpful to envisage new strategies for improved cardiac repair/regeneration. In this review, we provide a comparative synopsis of the major signaling pathways required for cardiac lineage commitment in the embryo and murine ESCs. The involvement and possible reactivation of these pathways following heart injury and their role in tissue recovery will also be discussed.

## 1. Introduction

Cardiogenesis relies on early specification of cardiac myocytes from mesodermal progenitors, incorporation of exogenous sources of precursors, and the spatial and timely integration of distinct signaling pathways. Genetic-based studies using the mouse embryo have uncovered regulatory cross-talks between distinct signaling pathways and a set of transcriptional cardiac regulators that control lineage commitment and heart morphogenesis. Additionally, embryonic stem cells (ESCs) derived from the inner cell mass of the embryo blastocyst have shown to constitute a powerful* in vitro* model that faithfully recapitulates the events occurring during embryo development. Similar to the embryo, mouse ESCs (mESCs) commit into the epiblast stage and undergo differentiation as aggregates designated embryoid bodies (EBs). These are able to differentiate into derivatives of the three germ layers in a sequential set of events that mimic embryo gastrulation (reviewed in [1]). Even though there are discrepancies in the timing of lineage progenitor segregation, once the cardiac molecular program is initiated, ESCs-derived cardiac progenitors engage in the recapitulation of all cardiac cell phenotypes, though no particular spatial organization is respected (reviewed in [2]). Thus, embryos and ESCs have been used in parallel to achieve increased understanding of the complex developmental process. Knowledge withdrawn from developmental studies has been used to promote* in vitro* cardiac differentiation of ESCs and these have also brought valuable mechanistic information to embryonic studies (reviewed in [2, 3]). Additionally, several pieces of evidence have shown that detailed study of the processes regulating heart specification and formation provides important clues to attain a better comprehension of cardiogenic mechanisms and to envisage improved strategies towards cardiac regeneration. Over the following sections, we will focus on the molecular events regulating cardiac specification in both embryo and ESCs. We will also address the signaling pathways shown to be reactivated in the mammalian myocardium following injury and how they can be modulated/potentiated in order to improve cardiac repair in pathological stress.

## 2. Molecular Events in Cardiogenesis

**Figure 1:**
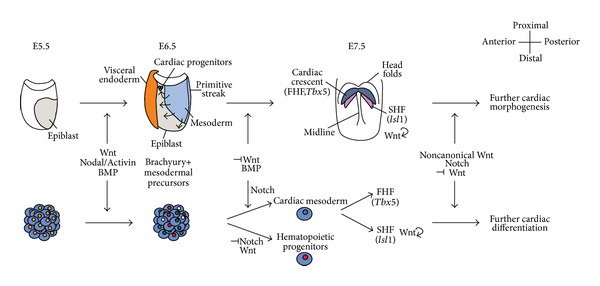
Signaling events in murine heart development and cardiac ESC differentiation. In both systems, mesodermal induction from the epiblast is regulated by Wnt/*β*-catenin, Nodal/Activin, and BMP signaling pathways and correlates with Brachyury upregulation. Further commitment of mesodermal progenitors to cardiac mesoderm and consequent first heart field (FHF) formation require the inhibition of Wnt signaling and expression of BMPs. Similarly, in ESCs system, Notch pathway inhibits Wnt/*β*-catenin signaling and activates BMP to specify cardiac fates. Wnt/*β*-catenin signaling is then activated to allow proliferation and maintenance of the SHF, both in embryo and ESCs. Further differentiation from the cardiac crescent stage to the following morphogenic phases of embryonic heart development and, in parallel, the expression of cardiomyocyte differentiation genes in ESCs require inhibition of Wnt/*β*-catenin. In the embryo and ESCs, this is achieved by Notch and noncanonical Wnt signaling, which inhibit the effect of Wnt/*β*-catenin and instruct progenitor cells within the SHF to leave the proliferative state and start differentiating. ⊣ represents inhibitory effect; ⤾ represents maintenance of a proliferative state.

In embryo development, gastrulation is a key event through which the three germ layers (endoderm, mesoderm, and ectoderm) are formed. Cellular fates are specified during gastrulation by both time of recruitment to the primitive streak (PS) and perceived morphogenetic information [4, 5]. Mesodermal induction is regulated by the interaction of distinct signaling pathways including bone morphogenetic proteins (BMPs), Nodal/Activin, and Wnt (reviewed in [6]). Mesodermal cells ingressing through the PS express the T-box transcription factor* brachyury* (*Bry*, also* T*), a direct target of the Wnt pathway [7]. *β*-catenin, a central player in Wnt signaling, has been shown to be essential for mesoderm formation since in *β*-catenin deficient mice no mesodermal or head structures are formed and* Bry* is not expressed [8]. These early embryonic events are also observed in ESCs, in which mesodermal commitment is defined by the upregulation of the* Bry* gene within 48 hours after the onset of differentiation (Figure 1) [9]. Mesoderm is then patterned and specified to originate distinct mesodermal subsets, characterized by differential expression of fetal liver kinase-1 (*Flk1*, also* Vegfr2*) and platelet-derived growth factor receptor-alpha (*Pdgfra*, also CD140a) [10]. Concomitantly,* Bry* expression in these cells decreases [11] and other transcription factors are activated. One key gene in both mouse embryo and mESCs is mesoderm posterior 1 (*Mesp1*) that has been correlated with definite cardiac commitment by activating the cardiogenic transcriptional network in a context-dependent manner (reviewed in [12, 13]). The conjunction of knowledge acquired from studying embryonic development and ESCs system led to the optimization of chemically defined cocktails that efficiently drive ESCs differentiation in the absence of serum (reviewed in [1]). Different studies have demonstrated that a tight balance between canonical Wnt and members of the transforming growth factor-*β* (TGF-*β*) superfamily, including Nodal/Activin and BMP signaling pathways, regulates the specification of the anterior and posterior regions of PS in mouse [14, 15] and human ESCs [16]. In fact, the combination of Activin A and BMP4 has been shown to direct mESCs into a mesodermal fate [17] whereas inhibition of the Nodal/Activin pathway drives human ESCs (hESCs) towards a neuroectoderm path [18]. Balanced levels of Nodal and BMPs determine mesoderm patterning: increased levels of Activin A favor FLK1^+^PDGFR*α* ^+^ cardiogenic progenitors while high doses of BMP4 promote the FLK1^+^PDGFR*α* ^−^ hematopoietic reservoir [19]. Importantly, activation of Notch pathway in differentiating mESCs has been shown to block the emergence of FLK1^+^ mesodermal progenitors [20].

Migration and specification of the primitive cardiac progenitors occur during gastrulation around mouse embryonic day (E) 6.5, when cells leave the PS and acquire an anterior-lateral position forming two groups of cells on both sides of the midline [4]. The presumptive cardiac cells, which will contribute to the myocardium and endocardium, can then be detected as a crescent in the mesoderm underlying the head folds (cardiac crescent, E7.5) (Figure 1). The crescent fuses at midline forming the beating primitive cardiac tube (E8), which subsequently folds to the right creating an S-shaped structure. The folded tube then suffers a series of rearrangements and cell expansion, which ultimately lead to the formation of recognizable septated cardiac chambers (E14.5). The cellular and morphogenic events underlying mammalian heart formation have been extensively reviewed elsewhere [5, 6, 21, 22]. At least two populations of mesodermal progenitors, arising from a common origin [23], partake in heart formation. The earliest group of progenitors (first heart field, FHF) constitutes the cardiac crescent and will contribute to the left ventricle (LV) and atria. FHF expansion depends on the second heart field (SHF) and provides a platform for subsequent heart growth. Cells of the SHF will form the outflow tract and the right ventricular region. Over the last years, transcriptional regulators directing the genetic program and morphogenesis of the cardiac progenitors have been uncovered: specific markers are still lacking for FHF (although* Tbx5* has been associated with this field), whereas* Isl1* has been considered a marker for SHF (reviewed in [6, 21]).

Both lineages of progenitors are regulated by a complex signaling network, emanating from the adjacent tissues. Similar to the embryo, evidences from the ESCs system suggest the existence of two cardiac fields or lineages with comparable molecular interregulatory networks (Figure 1) (reviewed in [2]). In the embryo, precardiac mesoderm induction and consequent FHF formation require ectodermal inhibitory Wnt signaling, as indicated by the respecification of endoderm into precardiac mesoderm in *β*-catenin depleted mice, resulting in the formation of several heart primordia [24]. These authors further observed ectopic expression of* Bmp2* following Wnt/*β*-catenin inhibition, suggesting that BMP signaling activation and Wnt inhibition are required to induce cardiac mesoderm specification. A similar regulation was observed using a Notch-inducible mESC line, in which Notch was capable of redirecting the hemangioblast into a cardiac fate through activation of BMP and inhibition of canonical Wnt pathways [25]. Accordingly in chick, zebrafish, and* Xenopus*, it has been shown that heart formation is induced in embryonic regions with high BMP2 and low Wnt activities (reviewed in [26]). The function of BMPs in the mouse appears to be more complex:* Bmp2*,* Bmp4*,* Bmp5*, and* Bmp7* are expressed in the anterior mesoderm, including the heart-forming regions but deletion of BMPs seems to have a late effect on cardiogenesis: mutants present cardiac defects and are embryonic lethal but cardiac mesoderm specification still occurs [2, 27, 28]. In conditional knockouts for* Bmpr1a*, encoding the BMP type 1 receptor, progenitors fail to progress towards specific lineages and form the cardiac tube [29]. Correct tissue specification relies not only on protein interconnections but also on the time of the signaling. For example, Wnt/*β*-catenin signaling presents a biphasic function in cardiogenesis: it has an inhibitory effect in the FHF but plays an inductive function in SHF proliferation. *β*-catenin inactivation in the SHF leads to defects in development and expansion of the SHF derivatives due to decreased cell proliferation, probably owing to reduced* Fgf10* levels (which promote SHF expansion) and residual* Bmp4* expression (capable of maintaining the antiproliferative effect of BMPs) [30]. *β*-catenin gain-of-function, on the other hand, leads to increased proliferation. This work shows a clear cross-talk between signaling pathways and evidentiates the crucial role of Wnt in regulating proliferation of cardiac progenitors within the SHF and maintenance of their undifferentiated state prior to entering the heart tube. The same activity for Wnt/*β*-catenin was described in ESCs since the use of a Wnt3a secreting feeder layer or conditioned media promotes expansion of ISL1^+^ progenitors and beating EBs whereas the addition of dickkopf-1 (DKK1), a canonical Wnt inhibitor, has the opposite effect, drastically reducing ISl1^+^ cells and beating EBs [31, 32]. Similar to *β*-catenin gain-of-function, Notch1 deletion in embryos and ESCs leads to augmented proliferation of cardiac progenitors through increased Wnt/*β*-catenin activity [33]. However, the mutant embryos failed to populate the developing right ventricle (which derives from ISL1^+^ cells) and genes associated with cardiomyocyte differentiation were downregulated in Notch-depleted ESCs. These observations suggest that Wnt/*β*-catenin inhibition is required to instruct progenitors to leave the SHF proliferative state and to start differentiating. Moreover, noncanonical Wnt pathway was also implicated in regulating SHF progenitors differentiation: loss of Wnt5a and Wnt11 affects SHF differentiation by increasing *β*-catenin nuclear levels [34]. The same authors [34] further showed that* Wnt5a* and* Wnt11* are required to promote cardiogenesis and induce the expression of cardiac-associated genes in differentiating ESCs, indicating that noncanonical Wnt signaling regulates the formation of FHF and SHF associated progenitors during EBs differentiation [34]. Additionally, exogenous noncanonical Wnt2 was shown to increase cardiomyocytic differentiation from murine ESCs [35]. Together, these results indicate that Notch signaling and noncanonical Wnt are required to instruct progenitors to leave the SHF proliferative state by interfering with Wnt/*β*-catenin pathway (Figure 1). Different members of the Notch pathway have been shown to be expressed along heart development regulating distinct key events in cardiogenesis. Notch proteins in the endocardium are responsible for modulating myocardial signals (e.g., BMPs) to regulate trabecular formation, chamber specification, and cell proliferation and differentiation (reviewed in [36, 37]). Neural crest cells and the proepicardial organ also contribute to the forming heart. Events such as cellular contribution from external sources, progenitor expansion, and differentiation towards the distinct cardiac cell types are tightly coordinated by the distinct signaling pathways, including Wnt, FGF, BMPs, Notch, and Hedgehog (Hh) (reviewed in [5]). These ultimately regulate a plethora of transcription factors that constitute a combinatorial code responsible for orchestrating cardiac development and specification and differentiation of myocytes.

## 3. Reactivation of the Embryonic Program in Cardiac Pathological Stress

The adult heart presents robust plasticity and it is capable of remodeling in response to distinct demands, either physiologic (normal aging or increased effort) or pathologic (e.g., hypertension, ischemia/myocardial infarction (MI) associated with coronary artery disease, hypertrophy, and dilated cardiomyopathies). In both cases, the first response to overcome the increased stress on the left ventricle (LV) is myocardial hypertrophic growth, which in the long-term is associated with increased risk of heart failure and sudden death (reviewed in [55, 56]). Efforts have been made towards identifying efficient new therapies to avoid heart failure. To accomplish this, a comprehensive understanding of the biological processes and signaling pathways involved in cardiac formation and leading to heart disease is required. Distinct diseases impacting the adult myocardium have been correlated with perturbation in signaling pathways involved in embryonic heart development. On the other hand, when under pathological stress, the heart reactivates pathways traditionally associated with the developing heart and whose activity is decreased in adult hearts (reviewed in [56, 57]). In this section, a general overview on the involvement of key pathways in heart disease will be presented (see also Table 1).

### 3.1. Notch Signaling

Being such an important signaling network in distinct mechanisms, perturbation of the Notch pathway has been associated with several genetic diseases and malformations. Regarding cardiac morphogenesis, Notch plays a crucial role in regulating events such as cardiomyocytes (CMs) differentiation, atrioventricular canal development, regulation of the endocardium endothelial-to-mesenchymal transition required both for valve formation and trabeculae development (reviewed in [36, 37]). Notch pathway is active in proliferating embryonic CMs but its activity decreases after birth and declines with age coinciding with CMs maturation [40, 58]. Furthermore, Notch activation in neonatal or mESCs-derived quiescent CMs induces cell cycle reentry [59]. The involvement of Notch in mammalian cardiac response to stress has been shown to be primarily mediated by notch1 and its ligand jagged1, which (together with the Notch target* Hes1*) are upregulated in the hypertrophic heart [38]. The authors also analyzed mice lacking* Notch1* specifically in the heart, which revealed increased hypertrophy, fibrosis, and mortality. Following MI,* Notch1* expression is also reactivated and detected in border zone CMs and this activation was correlated with repair and prosurvival processes, including prevention of CMs apoptosis, regulation of resident cardiac progenitor cells (CPCs) and immature CMs, and promotion of neovascularization ([40, 41, 60]; reviewed in [61]). Accordingly, delivery of the Notch intracellular domain (NICD) or of a notch1 pseudo-ligand following MI leads to improved wall thickness and cardiac function, enhanced neovascularization, and decreased infarct area [40, 41]. Overexpression of jagged1 in CMs restrains myocardial hypertrophy and fibrosis and promotes CPCs proliferation [42]. Importantly, inhibition of notch1 signaling with *γ*-secretase upon MI impairs the commitment of heart resident CPCs into the myocytic lineage [39]. This is of particular interest considering that stem/progenitor cells have been shown to contribute to generation of new CMs after injury, though they do not seem to actively participate in cardiomyocytic renewal during normal aging [62]. Taken together, these studies evidentiate Notch as an essential pathway with cardioprotective role in the damaged myocardium, being able to favor a procardiogenic process by regulating key events in cardiac remodeling as fibrogenesis and cardiogenesis.

### 3.2. FGF Signaling

FGFs are potent mitogens expressed from early development in the SHF, where* Fgf8* and* Fgf10* have been implicated in regulating progenitors proliferation and development together with other signaling pathways (reviewed in [5]). Expression of these growth factors is augmented during the onset of myocardial ischemia or infarction; their therapeutic potential has been addressed in pigs and dogs and shown to improve blood flow and preserved cardiac function in acute MI (reviewed in [63]). In rats, a combined treatment with FGF1 and p38 MAP kinase inhibitor following MI results in preserved wall thickness, reduced scaring, and overall improved cardiac function [43]. These effects are associated with increased proliferation and angiogenesis. FGF1* per se* is capable of inducing CMs cell cycle reentry and angiogenesis, but the combined therapy with p38 inhibitor enhances FGF effects and cardiac regeneration [43]. The role of FGF2, another potent angiogenic and mitogenic factor, in cardiac injury has also been extensively explored and shown to exert a protective effect against myocardial dysfunction following myocardial ischemia or infarction by increasing myocyte viability (reviewed in [44]).

### 3.3. Sonic Hedgehog Signaling

Sonic Hedgehog (SHH) morphogens are involved in several developmental processes during embryogenesis. In the heart, SHH ion (reviewed in [5]). Similar to other signaling pathways, there is evidence for* Shh* reactivation with concomitant upregulation of the Hedgehog patched1 (*Ptch1*) receptor in the ischemic myocardium [45]. In this study, the authors performed intramyocardial gene transfer of naked DNA encoding human* Shh*, which resulted in successful restoration of LV function in acute and chronic ischemia, enhanced neovascularization, and reduced fibrosis and apoptosis [45]. Interestingly, in a recent study, a strategy for controlled release of SHH morphogens was developed, which enables a slow and sustained delivery of SHH-heparin complexes, maintaining a constant local concentration within the therapeutic range [46]. This approach allowed a continued exposure of the myocardium to SHH, thus promoting a prolonged beneficial effect, which includes production of survival factors and attenuation of cardiomyocytic apoptosis [46]. These studies indicate that SHH treatment offers a putative therapeutic approach in acute and chronic ischemia.

### 3.4. Wnt Signaling

Different studies have shown that several Wnt factors are induced after experimental MI in various animal models, being involved in hypertrophy and cardiac wound healing following injury [64, 65]. Overall, blockage of Wnt signaling by targeting distinct pathway elements has a beneficial effect on cardiac remodeling (reviewed in [66, 67]). For example, the use of secreted frizzled-related proteins (SFRPs) that antagonize Wnt signaling by competing for Wnt binding and preventing ligation to the frizzled receptor reduces infarct size and improves cardiac function. This was shown either by inducing MI in transgenic mice overexpressing* Sfrp1* [47] or by SFRP2 local secretion [48] or exogenous administration [49]. SFRP2 was shown to increase myocardial survival after MI by preventing CM apoptosis and exerting an antifibrotic effect through* Bmp1* inhibition, normally involved in collagen biosynthesis [48, 49]. These and other reports (reviewed in [66]) seem to indicate a reactivation of the developmental mechanisms observed in FHF, in which Wnt inhibition is required for correct formation of the LV. In accordance, mice with cardiac-specific overexpression of dishevelled (*Dvl*), a protein acting downstream of frizzled receptor and activator of the canonical and noncanonical Wnt pathways, present myocardial hypertrophy and severe cardiomyopathy [50]. It is worth mentioning that, although the majority of reports indicate a beneficial effect upon inhibition of this pathway, some studies have demonstrated favorable outcomes upon its activation [67]. These differences might partially be due to variations in animal models, cell type, temporal context (essential for Wnt-mediated effects, as observed in embryonic heart development), and activation of Wnt-independent mechanisms by SFRPs [64, 68].

### 3.5. TGF/BMP Signaling

In cardiac embryo development, BMP signaling has been associated with valve formation:* Bmp2* deletion in the atrioventricular murine myocardium demonstrated that this protein is required for cardiac jelly formation and cardiac cushions development [69]. Conditional* Bmp4* mutants have profound defects in outflow tract and ventricle septation and perturbed expansion and remodeling of the endocardial cushions, resulting in abnormal valve structure [70]. In accordance with this embryonic role, TGF-*β* and BMPs in particular have been extensively implicated in valvular heart diseases in mammals and activated BMP signaling has been detected in diseased human aortic valves (reviewed in [71]). Accordingly, perturbing the endogenous repression of the BMP signaling cascade by deleting either the inhibitory SMAD6 [51] or noggin [52] leads to hyperplastic cardiac cushions due to increased cell proliferation. Besides the role in valve formation, TGF-*β* signaling is increased in stressed myocardium, being associated with augmented fibrosis and hypertrophic growth of CMs. Smad proteins, transcription factors downstream of TGF-*β*/BMP, positively regulate cardiac fibrosis, a major contributor to adult heart disease and functional impairment by regulating the expression of distinct extracellular matrix (ECM) proteins (reviewed in [56]). This was demonstrated to occur both in normal aging hearts and following MI. An increase in TGF-*β*1, SMAD proteins, and collagens was observed in infarcted rat hearts [72]. Regarding aging, 24-month-old* Tgfb1* heterozygous mice exhibited decreased myocardial fibrosis and stiffness when compared to control animals [73]. Additionally,* Tgfb1* overexpression induces cardiac hypertrophy, expression of hypertrophy-associated proteins, and increased connective tissue and interstitial fibrosis [54]. More recently, it was shown that inhibition of* Bambi* (BMP and Activin membrane-bound inhibitor), a negative regulator of TGF-*β*-mediated deleterious remodeling signals, leads to exacerbated hypertrophy, chamber dilation, deterioration of LV systolic function, and diastolic dysfunction [53].

## 4. Conclusions

Heart failure is a major concern in modern society. The approaches currently taken to achieve heart function restoration aim to delay or even reverse maladaptive remodeling. Even though several advances have been made, these strategies still face challenges like preservation of the contractile function and myocyte viability. We have reviewed distinct studies showing that in response to pathologic stress there is partial reactivation of genes that promote embryonic and fetal heart development. For example, Notch signaling may be modulated to expand the resident cardiopoietic progenitor pool and reactivate cell cycle reentry of preexisting cardiomyocytes in the adult mammalian heart in the scenario of pathological insult, limiting the extent of ischemic injury [39, 62]. Additionally, Shh holds great promise for repair/regeneration of tissues suffering ischemic injury, even though clinical translation has been hampered by its short half-life in the body [46]. Conversely, inhibition of Wnt/frizzled signaling pathway seems also to have beneficial effect on cardiac remodeling (reviewed in [66, 67]). In this sense, learning from the embryonic development can provide important clues to understand and modulate the injury scenario. This knowledge may be used in the future to implement and adopt new therapeutic strategies for adult heart disease. Interestingly, considering that resident CPCs have been shown to contribute to the generation of new cardiomyocytes in an injury setting [62], it would be valuable to analyze whether these signaling pathways are active in adult CPCs. In fact, Notch1 has already been shown to regulate adult CPCs proliferation and commitment to myocytes [39]. Furthermore, a strategy combining CPCs delivery with FGF controlled release is currently under clinical investigation [74]. These studies suggest that important pathways for embryonic cardiac morphogenesis can be translated to the adult signaling networks. One might then predict that the manipulation of this signaling environment will bring forward insights on how to modulate/potentiate CPCs response in a disease setting by creating a more suitable environment for repair/regeneration.

## Figures and Tables

**Table 1:** Overview of studies targeting different signaling pathways in heart pathological stress.

Pathway	Affected member	Effect	References
Notch	notch1 (⊣)	Increased hypertrophy, fibrosis, and mortality; impaired adult CPCs commitment into myocytic lineage	[38, 39]
	notch1 (→)	Improved wall thickness and cardiac function; enhanced neovascularization; decreased infarct area	[40, 41]
	jagged1 (→)	Restraint of myocardial hypertrophy and fibrosis; increased CPCs proliferation	[42]

FGF	FGF1 (→) FGF2 (→)	Preserved wall thickness; reduced scaring; improved cardiac function; increased proliferation and angiogenesis; increased CM viability	[43, 44]

SHH	*Shh* (→)	Restoration of LV function in acute and chronic ischemia; enhanced neovascularization; reduced fibrosis and apoptosis	[45]
	SHH-heparin complexes (→)	Production of survival factors; attenuation of CM apoptosis	[46]

Wnt/*β*-catenin	*sFrp1* (→)	Prevented CM apoptosis; antifibrotic effect	[47–49]
	SFRP2 (→)		
	dishevelled (→)	Myocardial hypertrophy; severe cardiomyopathy	[50]

TGF/BMP	SMAD6 (⊣)	Increased cell proliferation; hyperplastic cardiac cushions	[51, 52]
	noggin (⊣)		
	*Bambi* (⊣)	Hypertrophy; chamber dilation; deterioration of systolic function; diastolic dysfunction	[53]
	*Tgfb1* (→)	Cardiac hypertrophy; increased interstitial fibrosis	[54]

(⊣) Inhibition or (→) activation of the specific pathway member.
